# Supplementary material for: Content-rich biological network constructed by mining PubMed abstracts
Source: BMC Bioinformatics. 2004 Oct 8;5:147. doi: 10.1186/1471-2105-5-147 (PMC528731; doi:10.1186/1471-2105-5-147)
Supplement: Additional File 2 — The original results of the above study (non-essential files are deleted to keep the file size under the limit set by BMC bioinformatics). [file 1471-2105-5-147-S2.bz2 › chilibotAdditionalFile2/dip20/384ID1375038E9797/html/NTRK2_BDNF.html]

 


 **NTRK2** and **BDNF** 
  
Found 857 abstracts in PubMed, retrieved 20.  
 

 What does Google say? 
 PDF only 
| .edu only 

---

**Interactive relationship** (e.g. stimulation, inhibition, etc)

**Stimulatory relationship**- Moreover, three types of TrkB  [ **NTRK2** ]  dimers were induced by stimulating with exogenous  **BDNF** .  Ref: 12706205 J Chem Neuroanat, 2003
- Recent experimental evidence supports the role of  **BDNF**  in memory processes Memory acquisition and consolidation are associated with an increase in  **BDNF**  mRNA expression and the activation of its receptor TrkB  [ **NTRK2** ] .  Ref: 12719654 J Pharmacol Sci, 2003
**Neutral relationship**- **BDNF**  binds to the receptor tyrosine kinase TrkB  [ **NTRK2** ]  and transmits neurotrophic signals by activating neuron specific tyrosine phosphorylation pathways.  Ref: 12803487 Mol Cells, 2003
- Furthermore, recent findings suggest the importance of interaction between  **BDNF**  TrkB  [ **NTRK2** ]  signaling and NMDA receptors for spatial memory.  Ref: 12719654 J Pharmacol Sci, 2003
- These observations suggest that the functions of  **BDNF**  may be modified by interaction among subtypes of TrkB  [ **NTRK2** ]  in each region of the Purkinje cells.  Ref: 12706205 J Chem Neuroanat, 2003
- **BDNF**  then binds to and activates its cognate receptor, receptor tyrosine kinase B TrkB  [ **NTRK2** ] .  Ref: 12853306 Ann N Y Acad Sci< MedlineTA>Ann N Y Acad Sci, 2003
- The upregulation of  **BDNF**  expression, in turn, produced neuroprotective signals through receptor tyrosine kinase B TrkB  [ **NTRK2** ]  and promoted cell survival under the conditions of oxygen and glucose deprivation.  Ref: 12738784 J Biol Chem< MedlineTA>J Biol Chem, 2003
- Brain derived neurotrophic factor  [ **BDNF** ]   **BDNF** , a neurotrophin, binds to and activates its specific receptor tyrosine kinase, TrkB  [ **NTRK2** ] .  Ref: 12809697 Neuroscience< MedlineTA>Neuroscience, 2003

**Non-interactive relationship** (e.g. studied together, co-existance, homology, etc.)

- To test this, we infused the cortex with an inhibitor of Trk receptor tyrosine kinases, anti TrkB  [ **NTRK2** ]  IgG1, anti  **BDNF** , and anti neurotrophin 4 5 antibodies.  Ref: 12736347 J Neurosci, 2003
- We found that 1 trk C, a receptor for neurotrophin 3 NT 3, is dominantly expressed in RCEC, but trk B  [ **NTRK2** ] , a receptor for brain derived neurotrophic factor  [ **BDNF** ] , is dominantly expressed in RAEC.  Ref: 12745089 Biochem Biophys Res Commun, 2003
- Furthermore, the basal level of dopamine in the nucleus accumbens was not affected by treatment with both  **BDNF**  and TrkB  [ **NTRK2** ]  antibodies.  Ref: 12809697 Neuroscience< MedlineTA>Neuroscience, 2003
- Using an immunocytochemical method, we examined the expression of  **BDNF**  and its receptor, TrkB  [ **NTRK2** ] , in the taste bud cells of the circumvallate papillae of normal mice and of mice after transection of the glossopharyngeal nerves.  Ref: 12703550 Arch Histol Cytol, 2003
- Our results suggest that overexpression of  **BDNF** , NT 3, trkB  [ **NTRK2** ]  and trkC in motoneuron areas neighboring the site of mechanical compression may represent compensatory changes in response to the compromised neuronal function at the level of compression,.  Ref: 12774239 Acta Neuropathol Berl , 2003
- To test this hypothesis, we compared the ability of LIGA20 and  **BDNF**  to activate TrkB  [ **NTRK2** ] .  Ref: 12829403 Neurotox Res, 2002
- **BDNF**  and its specific receptor TrkB  [ **NTRK2** ]  are concerned with synaptic plasticity as well as maintenance of the nervous system.  Ref: 12706205 J Chem Neuroanat, 2003
- trkB  [ **NTRK2** ]  expression and  **BDNF**  responsiveness in neuroblastoma cells can be induced by all trans retinoic acid RA.  Ref: 12808116 Lab Invest, 2003
- As the VMN undergoes sexual differentiation, we compared  **BDNF** , NGF, NT 3, and trkB  [ **NTRK2** ]  mRNA expression in the VMN in males and females at embryonic day 20 and PND 4, but found no differences between them.  Ref: 12824057 Brain Res Mol Brain Res, 2003
- In addition, similar to MC4R mutants, mouse mutants that expresses the  **BDNF**  receptor TrkB  [ **NTRK2** ]  at a quarter of the normal amount showed hyperphagia and excessive weight gain on higher fat diets.  Ref: 12796784 Nat Neurosci< MedlineTA>Nat Neurosci, 2003
- To determine which of the neurotrophins NTs nerve growth factor NGF, brain derived neurotrophic factor  [ **BDNF** ]   **BDNF** , neurotrophin 3 NT3, and neurotrophin 4 5 NT4 and their receptors NTrs, TrkA, TrkB  [ **NTRK2** ] , TrkC, and p75, are present in the adult rat lacrimal gland.  Ref: 12882781 Invest Ophthalmol Vis Sci, 2003
- The present study was designed to investigate roles of  **BDNF**  and TrkB  [ **NTRK2** ]  in the expression of methamphetamine induced dopamine release in the nucleus accumbens and dopamine related behaviors induced by methamphetamine in rats.  Ref: 12809697 Neuroscience< MedlineTA>Neuroscience, 2003
- These findings provide further evidence that  **BDNF**  TrkB  [ **NTRK2** ]  pathway is implicated in the methamphetamine induced release of dopamine and the induction of dopamine related behaviors.  Ref: 12809697 Neuroscience< MedlineTA>Neuroscience, 2003
- For this purpose, serial immunohistochemical and immunoblot analyses were performed for the expression levels of endogenous brain derived neurotrophic factor  [ **BDNF** ]   **BDNF** , neurotrophin NT 3, and their receptors, trkB  [ **NTRK2** ]  and trkC in 24 tip toe walking Yoshimura mice twy twy aged 12 24 weeks.  Ref: 12774239 Acta Neuropathol Berl , 2003
- **BDNF**  in the taste bud cells may act as a neurotrophic factor for innervating sensory neurons through TrkB  [ **NTRK2** ]  receptors of the axons of those neurons, and also may exert autocrine and paracrine trophic actions on neighboring taste bud cells by binding to their TrkB  [ **NTRK2** ]  receptors.  Ref: 12703550 Arch Histol Cytol, 2003
- Genetic as well as pharmacologic deprivation of  **BDNF**  or TrkB  [ **NTRK2** ]  impairs learning and memory.  Ref: 12719654 J Pharmacol Sci, 2003
- LTD was induced when the  **BDNF**  TrkB  [ **NTRK2** ]  system was blocked.  Ref: 12736347 J Neurosci, 2003
- By 4 weeks after denervation, numerous TrkB  [ **NTRK2** ]  immunoreactive nerve fibers had invaded the papillae, and a few taste buds expressing  **BDNF**  and TrkB  [ **NTRK2** ]  had regenerated.  Ref: 12703550 Arch Histol Cytol, 2003
- In contrast, both the stimulation of dopamine release and induction of dopamine related behaviors by methamphetamine were significantly suppressed by pretreatment with intra nucleus accumbens injection of either  **BDNF**  2.0 & ; mgr ; l rat, 1 100 300 and 1 100 or TrkB  [ **NTRK2** ]  2.0 & ; mgr ; l rat, 1 1000 and 1 100 antibody.  Ref: 12809697 Neuroscience< MedlineTA>Neuroscience, 2003
- In contrast, the expression levels of  **BDNF** , NT 3, trkB  [ **NTRK2** ]  and trkC were significantly higher at the rostral and caudal sites immediately adjacent to the maximal compression site.  Ref: 12774239 Acta Neuropathol Berl , 2003
- We additionally observed the expression of  **BDNF**  and TrkB  [ **NTRK2** ]  in the developing circumvallate papillae of late prenatal and early postnatal mice.  Ref: 12703550 Arch Histol Cytol, 2003
- At P 0, a cluster of  **BDNF**  and TrkB  [ **NTRK2** ]  immunoreactive cells appeared in the dorsal epithelium of the papillae, and was presumed to be primitive taste buds.  Ref: 12703550 Arch Histol Cytol, 2003
- Cultured cortical progenitors express  **BDNF**  and NT 3, as well as their receptors TrkB tyrosine kinase  [ **NTRK2** ]  receptor B and TrkC.  Ref: 12832539 J Neurosci, 2003
- These findings suggest that  **BDNF**  TrkB  [ **NTRK2** ]  signaling in the hippocampus plays a crucial role in learning and memory.  Ref: 12719654 J Pharmacol Sci, 2003
- In this work the expression and anatomical localization of NGF,  **BDNF**  and NT 3 and of corresponding high affinity receptors TrkA, TrkB  [ **NTRK2** ]  full length and truncated TR isoforms, TrkC and of the low affinity p75 receptor were assessed in surgical samples from adult human lung by reverse transcriptase polymerase chain reaction RT PCR, Western blot and immunohistochemistry.  Ref: 12791675 Am J Respir Cell Mol Biol< MedlineTA>Am J Respir Cell Mol Biol, 2003
- Immunoreactivities for  **BDNF** , NT 3, trkB  [ **NTRK2** ]  and trkC were preferentially localized in the gray matter, particularly in the anterior horn cells.  Ref: 12774239 Acta Neuropathol Berl , 2003
- Administration of the other TrkB  [ **NTRK2** ]  ligand, brain derived neurotrophic factor  [ **BDNF** ]   **BDNF** , did not affect the normal course of the MyHC isoform transformation in this muscle, indicating that the observed effect is NT 4 5 specific.  Ref: 12771168 J Exp Biol, 2003
- Here we describe small, dimeric peptides designed to mimic a pair of solvent exposed loops important for the binding and activation of the  **BDNF**  receptor, trkB  [ **NTRK2** ] .  Ref: 12730196 J Biol Chem< MedlineTA>J Biol Chem, 2003
- Western blot analysis confirmed these results, showing that the lacrimal gland expresses NGF,  **BDNF** , NT3, and NT4 as well as the NTrs TrkA, TrkB  [ **NTRK2** ] , and TrkC and the p75 protein.  Ref: 12882781 Invest Ophthalmol Vis Sci, 2003
- This contrasts sympathetic neuroblasts, from which neuroblastomas are thought to arise, which neither express trkB  [ **NTRK2** ]  nor are dependent on the p145 trkB  [ **NTRK2** ]  ligands, brain derived neurotrophic factor  [ **BDNF** ]   **BDNF**  or neurotrophin 4 5, for their normal development.  Ref: 12808116 Lab Invest, 2003
- In contrast, null mutant mice for TrkB  [ **NTRK2** ] , the tyrosine kinase receptor for brain derived neurotrophic factor  [ **BDNF** ]  and neurotrophin 4, displayed a clear loss of locus coeruleus noradrenergic neurons.  Ref: 12810600 Development, 2003
- RT PCR showed positive bands at the appropriate sizes for NGF,  **BDNF** , NT3, and NT4, and for the receptors TrkA, TrkB  [ **NTRK2** ] , TrkC, and p75.  Ref: 12882781 Invest Ophthalmol Vis Sci, 2003
- the remaining taste bud cells still expressed  **BDNF**  and TrkB  [ **NTRK2** ] .  Ref: 12703550 Arch Histol Cytol, 2003
- Double labeling studies showed that  **BDNF**  and NCAM or TrkB  [ **NTRK2** ]  and NCAM colocalized in some of the taste bud cells, but that most taste bud cells were immunopositive for only  **BDNF**  or TrkB  [ **NTRK2** ] .  Ref: 12703550 Arch Histol Cytol, 2003
- Messenger RNAs encoding other neurotrophins, including nerve growth factor NGF and neurotrophin 3 NT 3 and the  **BDNF**  receptor trkB  [ **NTRK2** ] , were not selectively localized in neonatal VMN.  Ref: 12824057 Brain Res Mol Brain Res, 2003
